# Drivers of Microbial Carbon Fluxes Variability in Two Oligotrophic Mediterranean Coastal Systems

**DOI:** 10.1038/s41598-019-53650-z

**Published:** 2019-11-27

**Authors:** Natalia González-Benítez, Lara S. García-Corral, Xosé Anxelu G. Morán, Jack J. Middelburg, Marie Dominique Pizay, Jean-Pierre Gattuso

**Affiliations:** 1 Sorbonne Université, CNRS, Laboratoire d’Océanographie de Villefranche, 181 chemin du Lazaret, F-06230 Villefranche-sur-mer, France; 2 Institute for Sustainable Development and International Relations, Sciences Po, 27 rue Saint Guillaume, F-75007 Paris, France; 3 Department of Biology, Geology, Physics and Inorganic Chemistry, King Juan Carlos University, C/Tulipán s/n, 28933 Móstoles, Madrid Spain; 4 King Abdullah University of Science and Technology (KAUST), Red Sea Research Center, Biological and Environmental Sciences and Engineering Division, 23955-6900 Thuwal, Saudi Arabia; 5 Department of Estuarine and Delta Systems, NIOZ Royal Netherlands Institute for Sea Research and Utrecht University, Yerseke, The Netherlands; 6 Department of Earth Sciences, Utrecht University, Princetonlaan 8A, 3584 CB Utrecht, The Netherlands

**Keywords:** Microbial ecology, Marine biology

## Abstract

The carbon fluxes between phytoplankton and heterotrophic bacterioplankton were studied in two coastal oligotrophic sites in the NW Mediterranean. Phytoplankton and bacterial production rates were measured under natural conditions using different methods. In the Bay of Villefranche, the temporal variability revealed net heterotrophy in July-October and net autotrophy in December-March. The spatial variability was studied in the Bay of Palma, showing net autotrophic areas in the west and heterotrophic areas in the east. On average bacterial respiration, represented 62% of the total community respiration. Bacterial growth efficiency (BGE) values were significantly higher in autotrophic conditions than in heterotrophic ones. During autotrophic periods, dissolved primary production (DPP) was enough to sustained bacterial metabolism, although it showed a positive correlation with organic carbon stock (DOC). Under heterotrophic conditions, DPP did not sustain bacterial metabolism but bacterial respiration correlated with DPP and bacterial production with DOC. Temperature affected positively, DOC, BGE, bacterial respiration and production when the trophic status was autotrophic. To summarize, the response of bacterial metabolism to temperature and carbon sources depends on the trophic status within these oligotrophic coastal systems.

## Introduction

Marine biogeochemical carbon fluxes are largely variable and their temporal and geographical distribution are essential to understand the oceanic carbon cycle. The database of gross primary production, community respiration and net community production (NCP = gross primary production- community respiration) rates have increased considerably over the last years, but large gaps remain, especially for coastal oligotrophic areas^1–3^. In spite of their little area covering, approximately 7% of the surface of the global ocean, coastal waters account for 14% to 30% of total oceanic primary production^4^, which are highly variable in space and time and have a great ecological and social interest^5^. The metabolic balance or NCP informs us whether a marine ecosystem is net autotrophic (i.e. gross primary production > community respiration) or net heterotrophic (i.e. gross primary production < community respiration)^6,7^. Available data of oceanic primary production is mostly obtained from productive areas. However, there are orders of magnitude fewer measurements addressing in coastal oligotrophic regions, the carbon fluxes links between bacterial metabolism and the partitioning among dissolved (DPP) and particulate (PPP) primary production. Understanding how bacteria influence the cycling of organic carbon it is key to assess their role in the ecosystem metabolic balance^8^.

In oligotrophic Mediterranean coastal waters, it has been shown that large supply of dissolved organic carbon (DOC) from land can sustain elevated community respiration, resulting in net heterotrophy^9–11^. Autochthonous DOC, either released from normal algal growth or resulting from trophic interactions^12,13^, represent a large fraction (from 20 to 80%) of total primary production (DPP + PPP) in oligotrophic systems^14–16^. Indeed, the relative contribution of DPP to total primary production is considered an important autochthonous organic carbon source also for heterotrophic bacteria^17^, being considered by some authors as inverse^18^ or constant^19^ to the productivity of the system. Bacterial carbon demand includes all the organic carbon assimilated by heterotrophic bacteria, which can be allocated into anabolic (bacterial production) and catabolic processes (bacterial respiration**)**.

Currently, there is some debate about the dependence of bacterial carbon demand on phytoplankton production, mainly within oligotrophic systems^20,21^. In offshore areas not influenced by carbon inputs from land, phytoplankton primary production was reported to sustain bacterial carbon demand^18^, but the generality of this observation for inshore regions has not yet been confirmed^22^. Other authors^21^ suggested that bacterial carbon demand always exceeds contemporaneous primary production in all aquatic systems. Bacterial carbon demand is governed by bacterial growth efficiency (BGE = bacterial production/bacterial carbon demand), however there is a large range of variation in BGE (5–60%^23^;). In many instances, BGE is not directly measured but inferred from literature models^24,25^, which are generally biased with data from productive areas. Therefore, there is a lack of data about the BGE regulation in oligotrophic coastal waters^14,24,26–28^.

Ideally, direct measurements of net microbial carbon production and consumption should be used^29^ but bacterial production is usually measured in unfiltered samples and with short incubation times whereas bacterial respiration is measured in filtered samples with longer incubation times. In many studies bacterial respiration is derived from biomass and production data^30–32^. Moreover, disentangling the factors that control the carbon fluxes dynamics of marine microbial communities is challenging. The temperature dependence of biological processes has been traditionally expressed using the Van’t Hoff–Arrhenius equation also known as Boltzmann’s factor^33–35^. According to this expression, the slope of the natural logarithm of a metabolic rate against the temperature function 1/kT, where k is Boltzmann’s constant and T is the absolute temperature (Kelvin degrees), represents the activation energy (Ea) of that particular metabolic process^33–35^. Temperature has been described to play a major role regulating the metabolism of Mediterranean plankton communities^36,37^. Here we test how the metabolic status or balance of the plankton community can affect the temperature–dependence of microbial metabolism in these ecosystems.

**Figure 1:**
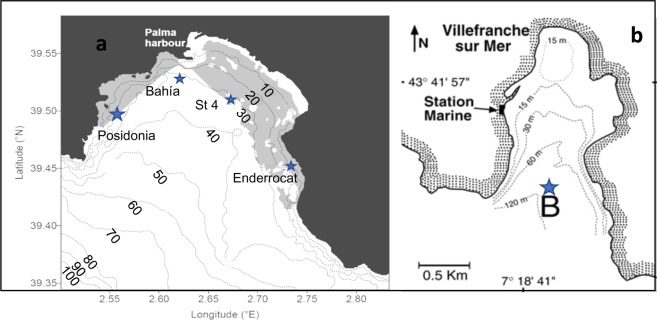
Study sites in (**a**) the Bay of Palma and (**b**) the Bay of Villefranche sur Mer. Stars show the location of the sampled stations.

To improve our knowledge of the trophic status in oligotrophic bays, it is necessary to focus the studies on temporal and spatial variability, despite the given idea that these environments as steady state systems. To reach this goal, we performed simultaneous measurements of net community production, particulate and dissolved primary production, bacterial production and respiration, thus allowing direct BGE, bacterial carbon demand and conversion factor values. The study was carried out in two oligotrophic coastal ecosystems in the NW Mediterranean (Fig. 1). The temporal variability was studied within the Bay of Villefranche (France) and the spatial variability was focused in July within the Bay of Palma (Spain), during the period most limited by nutrients. The comparison and join, of both bays is based on the environmental similarities of these regions^2,10^. The results will improve further understanding of the link between carbon fluxes and the metabolic trophic status, of these marine coastal ecosystems.

## Results

### Effect of filtration and incubation time on bacterial metabolism estimations

**Figure 2:**
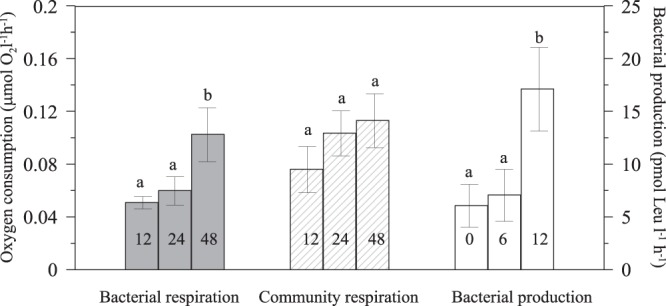
Effect of the incubation time under *in situ* simulated conditions on the respiration rates in filtered (bacterial respiration, BR) and unfiltered samples (community respiration, CR) and bacterial production rates (BP). Error bars represent standard error (n = 16). Lower case letters denote significantly different mean values (ANOVA, p < 0.05; Student-Newman-Keul post-hoc). Numbers within the bars indicate the time of incubation in hours.

To estimate the effect of filtration on bacterial metabolism, bacterial production, bacterial abundance and DOC concentration were compared between filtered and unfiltered samples. DOC concentration in unfiltered (83 ± 3 µmol l^−1^) and filtered (97 ± 5 µmol l^−1^) samples were significantly different (paired t-test; t_21_ = 3, p < 0.01, n = 22). Bacterial abundance was not significantly different before (6.92 ± 0.48 × 10^5^ cell ml^−1^) and after filtration (6.24 ± 1.02 × 10^5^ cell ml^−1^). Bacterial production was significantly lower in filtered (17.59 ± 4.03) than in unfiltered (66.45 ± 11.41) samples (paired t-test; t_83_ = 4.5, p < 0.001, n = 84). Bacterial production was estimated after different periods of *in situ* incubation times (no incubation, 6 and 12 h). After 12 h incubation bacterial production showed values 2.6-times higher compared with those incubated during shorter incubations (0 and 6 h) (F_2,45_ = 4.3, n = 48, p = 0.02; Fig. 2). Also, a significant effect of incubation time was observed in bacterial respiration rates (Fig. 2; F_2,21_ = 4.1, n = 24, p = 0.03), with higher values after 48 h than after 12 and 24 h (Fig. 2). However, the duration of incubation had no significant effect on unfiltered samples. BGE and bacterial carbon demand are usually estimated using bacterial production and respiration rates which are commonly incubated during different time lengths. In order to minimize possible bias, BGE_12_ and BCD_12_ in our experiments were estimated using bacterial respiration and production measurements from pre-filtered samples both incubated during 12 hours (see material and methods). The average percentage of oxygen consumption in filtered seawater samples (12 and 24 h) compared to whole seawater samples, were enabled to estimate bacterial respiration.

### Temporal variability in the Bay of Villefranche

**Figure 3:**
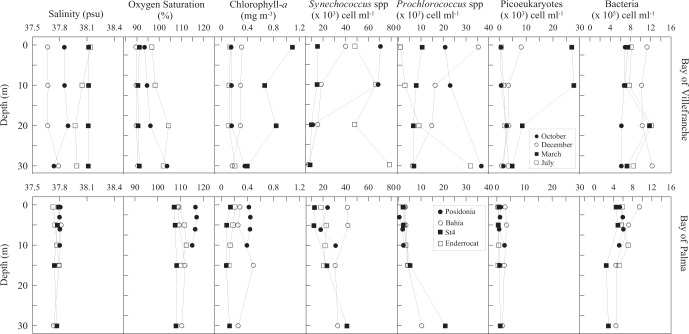
Vertical profiles of salinity (psu; practical salinity units), percentage of oxygen saturation, Chlorophyll-a (mg m^−3^), *Synechococcus* spp (x 10^3^cell ml^−1^), *Prochlorococcus* spp (x 10^3^cell ml^−1^), Picoeukaryotes (x 10^3^cell ml^−1^) and heterotrophic prokaryotes (x 10^5^cell ml^−1^) abundances in the Bay of Villefranche (upper panels) and Bay of Palma (lower panels). Error bars represent standard error.

As expected, irradiance showed a seasonal pattern with the highest values found during July followed by October, December and March (Table 1). The lowest temperatures were recorded in March (13.2 °C throughout the water column) and the highest in July, coincident with the strongest stratification (Table 1). Changes in the percentage of O_2_ saturation were similar to those of temperature, with oxygen-saturated waters during July and below 100% during the rest of the year (Table 1, Fig. 3). The concentrations of NO^−^_3_ and Si (OH)_4_ were lower than 1 µmol l^−1^ except in March (Table 1). The highest DOC concentrations were during July (70.7 ± 2.2 µmol l^−1^) and the lowest during March (63.8 ± 0.6 µmol l^−1^; Table 1).

**Table 1:** Average ± SE values within the photic zone for selected variables at the different periods and stations sampled.

	Sampling depths	Salinity	O_2_ Saturation	Irradiance	Temperature	Si (OH)_4_	NO^−^_3_	DOC	BGE_12_	PER
	m	psu	%	µmol photons m^−2^ s^−1^	°C	µmol l^−1^	µmol l^−1^	µmol l^−1^	%	%
**Bay of** **Villefranche**										
October	0.5, 10, 20, 30	37.8	96.8 ± 2.3	462.5 ± 277.8	20.7 ± 0.9	0.1 ± 0.0	0.9 ± 0.4	66.3 ± 1.0	0.6 ± 0.1	55.8 ± 7.2
December	0.5, 10, 20, 30	37.7	89.8 ± 0.2	261.7 ± 164.6	17.2 ± 0.0	0.8 ± 0.0	0.7 ± 0.2	64.4 ± 1.6	1.9 ± 0.7	20.4 ± 6.7
March	0.5, 10, 20, 30	38.1	90.7 ± 0.2	184.2 ± 72.2	13.2 ± 0.0	1.7 ± 0.4	3.0 ± 0.3	63.8 ± 0.6	0.5 ± 0.1	54.8 ± 12.8
July	0.5, 10, 20, 30	38.0	100.9 ± 1.7	675.0 ± 318.1	21.3 ± 2.2	0.7 ± 0.1	0.7 ± 0.2	70.7 ± 2.2	7.3 ± 5.1	31.6 ± 7.2
**Bay of Palma**										
Posidonia	0.5, 3, 6, 10	37.8	116.1 ± 0.4	581.7 ± 166.5	22.6 ± 0.1	0.4 ± 0.1	0.3 ± 0.0	89.1 ± 4.4	17.5 ± 8.1	36.5 ± 7.1
Bahia	0.5, 5, 15, 30	37.8	110.1 ± 0.7	495.9 ± 200.1	22.4 ± 0.7	0.9 ± 0.1	0.3 ± 0.0	85.5 ± 1.7	10.9 ± 3.3	36.6 ± 1.9
Station 4	0.5, 5, 15, 30	37.8	107.6 ± 0.2	478.4 ± 187.0	20.8 ± 0.7	1.0 ± 0.1	0.4 ± 0.0	71.6 ± 2.5	3.4 ± 1.1	61.2 ± 2.5
Enderrocat	0.5,5, 10, 15	37.8	110.8 ± 0.7	619.2 ± 170.5	23.0 ± 0.5	1.0 ± 0.0	0.3 ± 0.0	68.8 ± 4.2	1.1 ± 0.5	78.3 ± 3.2

Salinity, percentage of oxygen saturation, irradiance, temperature, silicate (Si (OH)_4_), nitrate (NO^−^_3_), dissolved organic carbon (DOC), bacterial growth efficiency (BGE_12_) and percent extracellular release (PER).

Chlorophyll-*a* concentrations varied considerably (from 0.1 to 1.1 mg m^−3^) across depths and months, with mean values increasing from summer to spring (Fig. 3). The picophytoplankton community composition was dominated by *Synechococcus* cyanobacteria. The highest abundances of *Synechococcus* were found in October and July, while *Prochlorococcus* peaked in December and October and photosynthetic picoeukaryotes in March. Heterotrophic prokaryotes abundances ranged from 6.1 to 12.3 × 10^5^ cells ml^−1^, with the highest abundances measured in December and the lowest in October (Fig. 3). Picoautotrophs biomass average contributed with 9.4 (±1.1) % of the total autotrophic biomass (Chl-*a*).

**Figure 4:**
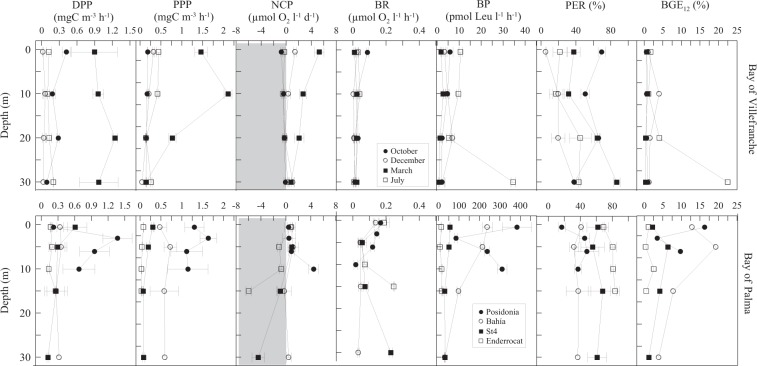
Vertical profiles of the rates of dissolved (DPP) and particulate (PPP) primary production (mg C m^−3^ h^−1^), net community production (NCP, µmol O_2_ l^−1^ d^−1^), bacterial respiration (BR, µmol O_2_ l^−1^ h^−1^) and bacterial production (BP, pmol Leu l^−1^ h^−1^). Also show the percent extracellular release (PER = DPP / [DPP + PPP]) and bacterial growth efficiency (BGE_12_, %) in the Bay of Villefranche (upper panels) and the Bay of Palma (lower panels). Error bars represent standard error.

Primary production was generally low, with DPP and PPP ranging from 0.02 to 1.36 mg C m^−3^ h^−1^ and 0.03 to 2.10 mg C m^−3^ h^−1^; respectively, with the highest values observed in the spring bloom period during March (Fig. 4). Percentage of extracellular release was highly variable, from 5.0 to 86.6%, with the lowest values recorded in December and the highest ones in October (Table 1, Fig. 4). Heterotrophic bacterial production showed a minimum of 1.6 ± 0.4 pmol Leu l^−1^ h^−1^ in March (equivalent to 0.002 mg C m^−3^ h^−1^) and a maximum of 14.9 ± 6.6 pmol Leu l^−1^ h^−1^ in July (0.02 mg C m^−3^ h^−1^). Indeed, bacterial respiration (BR) was lowest in December (0.01 ± 0.004 µmol O_2_ l^−1^ h^−1^) and highest in October (0.04 ± 0.01 µmol O_2_ l^−1^ h^−1^) (Fig. 4). BGE_12_ ranged from 0.3 to 22.4%, with the highest value in summer and lowest in spring (Table 1).

### Spatial variability in the Bay of Palma

Irradiance and temperature values were very similar between stations, with approximately 500 µmol photons m^−2^ s^−1^ and 22.2 °C (Table 1). Salinity averaged 37.8 psu and mean O_2_ saturation were above 100% saturation across sites (Table 1, Fig. 3). Low dissolved inorganic nutrient concentrations characterized these oligotrophic waters, with NO^**−**^_3_ and Si (OH)_4_ always below or equal to 1 µmol l^−1^ (Table 1). DOC concentration was higher at the western stations Posidonia and Bahia, (89.1 ± 4.4 and 85.5 ± 1.7; µmol l^−1^, respectively (Fig. 1, Table 1). Chorophyll-*a* values were below 0.5 mg m^−3^ and followed the same pattern as DOC with the highest values measured at Posidonia and Bahia stations (Fig. 3). Picophytoplankton community composition was dominated by *Synechococcus* spp across stations, with highest abundances measured at Bahia station (Fig. 3). *Prochlorococcus* spp peaked at Station 4 and photosynthetic picoeukaryotes at Bahia station. Heterotrophic prokaryotes abundance ranged from 2.4 to 9.4 × 10^5^ cells ml^−1^, with the highest abundances at Bahia and lowest at Station 4 station (Fig. 3). The average picoautotrophs biomass accounted 5.7% ( ± 0.7) of the total autotrophic biomass. Total primary production ranged from 0.16 to 2.99 mg C m^−3^ h^−1^, with DPP ranging from 0.11 to 1.36 mg C m^−3^ h^−1^ and PPP from 0.03 to 1.63 mg C m^−3^ h^−1^. Highest values of both fractions, DPP and PPP, were measured at Posidonia station and the lowest at Enderrocat (Fig. 4). Percentage of extracellular release averaged 53.4 ± 3.7%, with highest values observed at Enderrocat station and similarly low values at Posidonia and Bahia stations showing an opposite tendency of chlorophyll-*a* and DOC (Table 1, Fig. 4).

In the Bay of Palma, the lowest bacterial production was 11.8 pmol Leu l^−1^ h^−1^ (0.01 mg C m^−3^ h^−1^) observed at Enderrocat station and the highest in Posidonia station of 252.9 pmol Leu l^−1^ h^−1^ (0.29 mg C m^−3^ h^−1^) (Fig. 4). Bacterial respiration showed the lowest value at Bahia station, with 0.06 µmol O_2_ l^−1^ h^−1^ and the highest at Station 4 station with 0.24 µmol O_2_ l^−1^ h^−1^ (Fig. 4). BGE_12_ ranged from 0.3 to 40.6%, with the highest mean values at Posidonia station and the lowest observed at the Enderrocat station (Table 1).

### Effects of environmental and biological variables on bacterial metabolism on both bays

The temporal variability in the Bay of Villefranche included net heterotrophy during summer and autumn (−0.2 ± 0.2 and −0.4 ± 0.1 µmol O_2_ l^−1^ d^−1^, respectively) and net autotrophy during winter and spring (0.6 ± 0.4 and 2.7 ± 0.9 µmol O_2_ l^−1^ d^−1^, respectively). The spatial variability in the Bay of Palma communities showed autotrophy at the western stations Posidonia and Bahia (1.5 ± 1.0 and 0.5 ± 0.3 µmol O_2_ l^−1^ d^−1^, respectively) and heterotrophy, in the eastern stations Enderrocat and Station 4 (−1.8 ± 1.5 and −2.5 ± 1.5 µmol O_2_ l^−1^ d^−1^, respectively).

**Figure 5:**
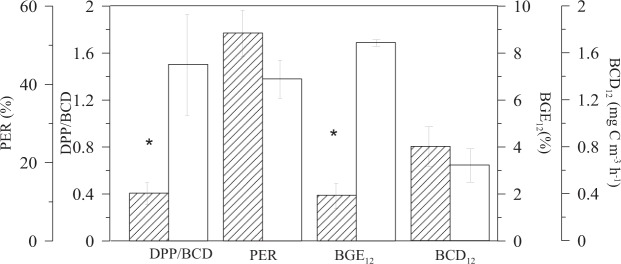
Effect of the trophic state (empty bars are autotrophic and dashed bars are heterotrophic) on Dissolved Primary Production /Bacterial Carbon Demand ratio (DPP/BCD), Percentage of Extracellular Release (PER), Bacterial Growth Efficiency (BGE_12_) and Bacterial Carbon Demand (BCD_12_). Error bars represent standard error. Significant differences are shown with an asterisk.

All data were grouped into two categories regardless of the location and from an ecophysiological point of view: net autotrophic (NCP > 0) and net heterotrophic (NCP < 0). Coherently, results confirmed that DPP and PPP were significantly higher in autotrophic relative to heterotrophic conditions (F_1,30_ = 7.1, n = 32, p = 0.01 and F_1,30_ = 10.8, n = 32, p = 0.002, respectively). DPP sustained bacterial carbon demand under autotrophic conditions (DPP/BCD = 1.5 ± 0.4), whereas it did not under heterotrophic (DPP/BCD = 0.4 ± 0.1; Fig. 5). BGE was also significantly higher (F_1,30_ = 5.1, n = 32, p = 0.03) in autotrophic than in heterotrophic communities (8.5 ± 2.6% *vs* 1.9 ± 0.5%) (Fig. 5). However, percentage of extracellular release and bacterial carbon demand showed no significant differences between trophic status; respectively (F_1,30_ = 1.7, n = 32, p > 0.05 and F_1,30_ = 0.5, n = 32, p > 0.05) (Fig. 5).

**Figure 6:**
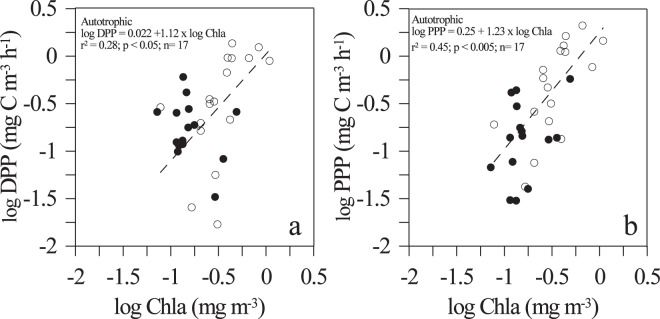
Relationship between Dissolved Primary Production (DPP) (**a**) and Particulate Primary Production (PPP) (**b**) with Chlorophyll-*a* concentrations under autotrophic (white dots) and heterotrophic (black dots) conditions. Dashed lines represent the fitted Model II linear regressions for the significant relationship within autotrophic samples.

When differentiated according to NCP values, DPP and Chl-*a* were positive and significant linearly correlated only within autotrophic communities (R^2^ = 0.28, n = 17, p = 0.03), but not within heterotrophic ones (p > 0.05; Fig. 6a). Similarly, the linear regression between PPP and chlorophyll-*a* was significant and positive for autotrophic communities (R^2^ = 0.45, n = 17, p = 0.003), whereas no correlation was found in the heterotrophic ones (p > 0.05; Fig. 6b).

**Figure 7:**
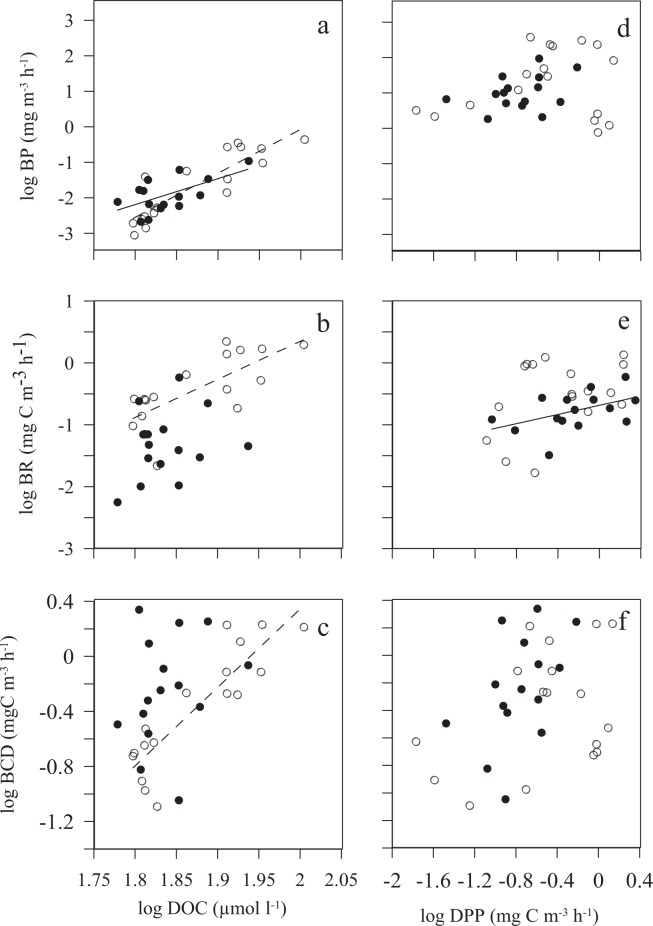
Relationship between: Dissolved Organic Carbon (DOC) and Dissolved Primary Production (DPP) with: Bacterial Production (BP) (**a,d**); Bacterial Respiration (BR) (**b,e**) and Bacterial Carbon Demand (BCD) (**c,f**). Black and white dots represent heterotrophic and autotrophic conditions, respectively. The solid line (for heterotrophic conditions) and dashed line (for autrotrophic conditions), represent the significant fitted Model II linear regressions. The equations parameters are: a) log BP = −24.87 + 12.40 × log DOC; r^2^ = 0.76; p < 0.0001; n = 17 (dashed line for autotrophic conditions). log BP = −15.34 + 7.30 × log DOC; r^2^ = 0.36; p < 0.05; n = 15 (solid line for heterotrophic conditions). (**b**) log BR = −11.94 + 6.14 × log DOC; r^2^ = 0.53; p = 0.001, n = 17 (dashed line for autotrophic conditions). (**c**) log BCD = −11.07 + 5.70 × log DOC; r^2^ = 0.77; p < 0.001, n = 17 (dashed line for autotrophic conditions). (**e**) log BR = 0.72 + 1.24 × log DPP; r^2^ = 0.50; p < 0.005, n = 15 (solid line for heterotrophic conditions).

Bacterial production showed a significant relationship with DOC concentration independently of the trophic status of the community (Fig. 7a) while bacterial respiration and carbon demand were correlated with DOC concentration only with autotrophic communities (Fig. 7b,c). In contrast, bacterial respiration was only significantly related to DPP in heterotrophic communities (Fig. 7e), whereas no relationships of bacterial production and carbon demand were observed with DPP (Fig. 7d,f).

**Figure 8:**
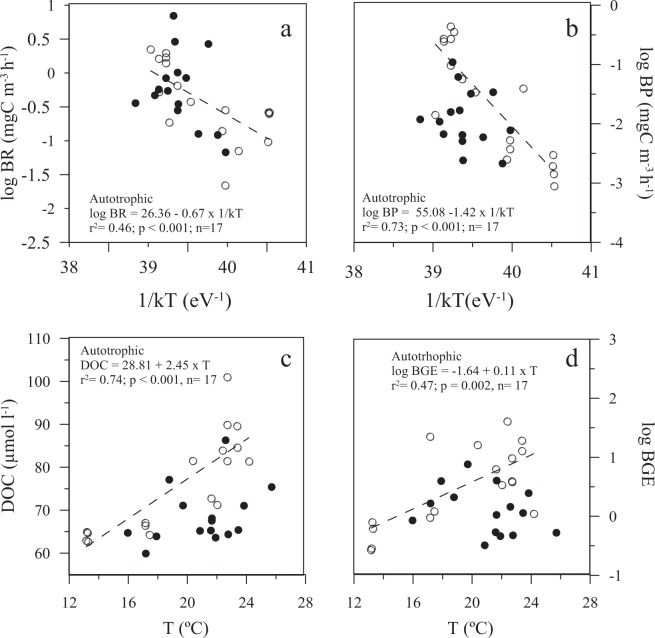
Temperature dependence relationship between log-transformed (**a**) Bacterial respiration and (**b**) Bacterial production *vs* water temperature (1/kT with k, the Boltzmann’s constant (8.617734 10^−5^ eV K^−1^) and T the water temperature (°K)). (**c**) Dissolved organic carbon (DOC) and (**d**) Log-transformed bacterial growth efficiency *vs* water temperature (°C). Black and white dots represent heterotrophic and autotrophic conditions, respectively. The dashed lines and equations parameters represent the significant fitted model II regressions.

Bacterial production and respiration, BGE and DOC concentrations were positively correlated with temperature only under autotrophic conditions, but not under heterotrophic (Fig. 8a–d). The slope or activation energy of the Arrhenius relationship for bacterial production was 1.42 eV, 0.67 eV for bacterial respiration and 0.83 eV for BGE.

## Discussion

Relying on actual system-specific measurements rather than on literature or models results, does not completely solve the coupling degree between phytoplankton and heterotrophic bacterioplankton because measuring the flow of carbon through bacteria is methodologically challenging. For instance, bacterial respiration and production estimates may still bring uncertainties due to factors such as the incubation time, bottle effects, bottle opaque to UVB radiation, filtration artefacts or the choice substrate-to-carbon conversion factors^29,37–39^.

Our first concern was to make sure that bacterial abundance was not significantly different after filtration, so that metabolic processes measured in the filtered samples represented realistically the rates of bacterial respiration in the no processed samples.

Filtering process by the 0.8 µm filters increases DOC concentration because some planktonic cells could have been broken, leaking part of their cytoplasmic content into the filtrate^40^. However, this process even if did not affect small bacteria it certainly had influence on attached bacteria^29,41^ which are not detected by the flow cytometer. For this reason, bacterial production was higher before the filtering process and heterotrophic prokaryotes abundance did not show any significant difference.

Since bottle effects are to some extent unavoidable (e.g.^42^), another factor contributing to uncertainty is the incubation time. Contrary to previous studies^27^, bacterial respiration and production did not show linear responses after 12 and 48 h, respectively. This observation is not trivial, as most of the previous studies did not consider as a key variable the incubation time or the bottle effect^14,36,43^. Bacterial production is typically measured in unfiltered samples and incubated for a short period (2–8 hours), whereas bacterial respiration is determined in filtered samples that are subsequently incubated for at least 24 or 48 h. Bacterial respiration could be stimulated during long incubations (48 h^42^) by viral activity^44^ or by changes in bacterial community composition^26,45^. Shorter incubations (i.e. <6 h) limit the bottle effect and provide the most accurate consumption rates of labile DOC^9^. On the contrary, samples incubated for longer times will be exposed to higher cumulative processes, such as cell lysis, increasing DOC release and consequently increased bacterial production and respiration^46^.

Despite the methodological challenge, in this study we were able to demonstrate coherent patterns in bacterial carbon fluxes in two NW Mediterranean embayment’s. Our direct rather than indirect estimations and the simultaneous temporal and spatial studies, might provide a new insight of the bacterial carbon fluxes and their temperature and metabolic status dependence within oligotrophic systems.

In this study, we measured an expected significantly higher chlorophyll-a concentrations under autotrophic periods (stations which values of NCP are higher than 0) compare to those concentrations under heterotrophic conditions (NCP < 0). However, picophytoplankton biomass of the diverse groups measured, was not significantly different during heterotrophic and autotrophic periods. Indeed, regardless to the locations TPP, and consequently DPP and PPP, was significantly higher under autotrophic conditions compare to heterotrophic conditions. Contrary to previous works^16,18^ and in agreement with others^19^ differences in percentage of extracellular release average values between trophic states were not significant (41 ± 5% and 53 ± 5% in autotrophic and heterotrophic systems, respectively).

Our results showed a robust relationship between DPP and PPP with photosynthetic proxy biomass (Chl-*a*) in autotrophic waters, while they were not correlated in heterotrophic waters. At the Bay of Villefrance-sur-Mer, maximum rates of DPP and PPP were registered during March when nitrate and chlorophyll-*a* concentrations were higher and seawater temperature was lower. This situation corresponds with the spring phytoplankton bloom and is followed by an increase in bacterial production as a consequence of increased DPP^19,47^. However, the lack of correlation between DPP and PPP with chlorophyll-*a* under heterotrophic conditions, may be attributable to a strong nutrient limitation for phytoplankton growth^48^, due to process such as grazing^16^ or to phytoplankton lysis^49^. Despite the general low nutrient concentrations measured, during the spring bloom of March, the high nitrate concentrations measured (>1 up to 4 µmol l^−1^) were similar to those registered in the same area^2^, but lower than other oligotrophic Mediterranean regions (up to 7.0 µmol l^−1^ for nitrate in^14^).

Contrary to the previous described situation^2,14,50^, during summer at the Bay of Palma, the lowest nitrate concentration co-occurred with elevated water temperature (23.4 °C) and high DOC concentrations (90 µmol l^−1^). Some authors^9,51^ reported an accumulation of DOC during summer and autumn and attributed this to the inability or inefficiency of bacteria to use it under nutrient limitations, the so-called malfunctioning microbial loop^52^.

However, not only DOC, but also phytoplankton DPP has been considered an important carbon source to support bacterial metabolism^18,19^. In our study, we observed a positive correlation between DPP and BR, but exclusively when the system was heterotrophic. Under heterotrophic nutrient-deplete conditions, the fresh and labile carbon from phytoplankton production (DPP) may be firstly respired for bacterial basal metabolism^53^. Although, under the same heterotrophic scenario, bacterial production is correlated to DOC concentrations instead of DPP, probably due to the extra resources needed for bacterial growth or production, than those used to maintain basal bacterial respiration^31^. Therefore, at resource limiting conditions when phytoplankton production was too low to sustain BP, labile DOC from nearby seagrass meadows may be used as an organic carbon source^54–56^. Since abundant beds of *Posidonia oceanica* were present in both bays, a large fraction of the DOC used for bacterial production may come from the exported carbon from benthic organisms^56,57^.

On the other hand, under autotrophic conditions, bacterial metabolism, was positively correlated with DOC concentrations and DPP was potentially sufficient to sustain the bacterial carbon demand (DPP/BCD > 1). These results suggest that DPP contributed signifiicantly to the labile DOC pool under autotrophic conditions and bacteria were growing actively when DOC stock concentration was higher enough to maintain bacterial metabolism^2,9,28,58,59^. The highest DPP/BCD ratio was found in Villefranche´s Bay during March when the spring bloom occurred and picoeukaryotes biomass was higher than other picoautotrophs groups. Although picoeukaryotes only accounted 3% of the total autotrophic biomass, concurred with the higher DPP concentrations measured^60,61^ during that period.

The previous reported BGE values depend, however, on the particular leucine-to-carbon conversion factor used^31^. In this work, bacterial production was estimated from *in situ* incubations and calculated with empirically determined leu-to-C conversion factors as emphasized in recent studies^27,62,63^. Bacterial production values (0.001 to 0.44 mg C m^−3^ h^−1^) were within the range reported in other Mediterranean coastal sites^14,36,64^, being more active during autotrophic periods, and leading to higher BGE values up to 4-fold compare to those periods where heterotrophic conditions dominated.

It has also been described that bacterial respiration contribute with a significant amount to total community respiration, frequently more than 50%^7,25,65^. In our study, bacterial respiration represented, on average, 62% of the total community respiration and did not depend on the metabolic balance of the system. However, bacterial respiration mean value at both coastal oligotrophic environments (1.9 ± 0.2 mmol C m^−3^ d^−1^) was substantially lower than the average for coastal regions (7.1 ± 12.2 mmol C m^−3^ d^−1^) given in the revision of Robinson^39^. Indeed, bacterial respiration was only slightly higher than open ocean value (1.3 ± 0.2 mmol C m^−3^ d^−1^), probably showing the general oligotrophic nature of Mediterranean coastal waters.

Our BGE values (0.3–40.6%) were within the range of published values for the Mediterranean Sea^2,14,36,58,66^. However, the median BGE (1.6%) was substantially lower than the value given for coastal regions (16%) and even the open ocean (8%)^39^, although it should be noted that this review^39^ did not include any data from the coastal oligotrophic Mediterranean.

Temperature, nutrient concentrations, dissolved organic matter lability and bacterial taxonomic composition have been regarded among the main factors that can influence BGE^14^. Robinson^39^ suggested that most of the BGE variability within oligotrophic waters is explained by bacterial respiration. Other authors^31^ found that BGE is not directly regulated by temperature, but by the availability of substrates for growth. According to our results, temperature explains a large part of BGE (47%), bacterial respiration (46%) and production variances (73%) when there is a not nutrient limitation. Indeed, the metabolic status of the system is a critical factor determining the temperature-dependence of bacterial metabolic rates. Consequently, the significant and positive relationships between temperature and bacterial metabolic rates (bacterial production and respiration, BGE) during periods of net autotrophy did not hold for heterotrophic periods. Our results confirm that substrate availability is a key factor governing the temperature dependence of heterotrophic bacterial metabolism^31,67^. Temperature is a relevant factor under autotrophic conditions, when our system is in the stage between the bottom-up and top-down control^68^. However, temperature is not a main driver of bacterial metabolism under heterotrophic conditions, due to the limited resource availability (bottom-up control).

The lower slope of the bacterial respiration regression (Ea_BR_ = 0.67 eV) indicates a lower activation energy required for this process relative to that of bacterial production (Ea_BP_ = 1.42 eV), what confirms their different temperature-dependence as an intrinsic characteristic of two independent biochemical processes^59^. 

The bacterial respiration activation energy value estimated in this study (Ea_BR_ = 0.67 eV) is very close to the theoretical Ea of 0.65 eV predicted by the Metabolic Theory of Ecology for respiratory processes^33,34^. Compared to previous works, our bacterial respiration Ea value is lower than the values reported by Mazuecos^69^ for the dark ocean (0.90 eV) and higher than those values for the global ocean (*Ea*_BR_ = 0.589 eV^31^), (*Ea*_BR_ = 0.57 eV^70^).

Bacterial production activation energy (Ea_BP_ = 1.42 eV) is more than twice the maximum value described by Morán^71^ (from −0.14 to 0.67 eV), although it is within the range described by Arandia-Gorostidi^72^ (−0.3 to 1.46 eV) both for the NE Atlantic coastal waters, and slightly higher than the *in situ* bacterial production measured at the Baltic Sea (1.24 ± 0.16 eV^73^). The relationship of bacterial metabolic rates with temperature under autotrophic conditions, agrees with higher Eas values during the highest nutrient concentration^71^ and productive bloom periods^67,72^.

We conclude that the metabolic status (autotrophic vs heterotrophic) of the oligotrophic coastal Mediterranean studied systems, determines the source of organic carbon where the bacterial communities are supported by, as well as the temperature dependence of microbial metabolic processes.

## Material and Methods

### Study site and sampling

The spatial variability was investigated in the Bay of Palma in June 2002 (Fig. 1a). Four stations were sampled: Enderrocat (39.4°N 2.7°E), Station 4 (39.5°N 2.4°E) in the east of the bay and Bahía (39.5°N 2.6°E) and Posidonia (39.5°N 2.5°E) located in the west. Stations Posidonia and Bahia are located above *Posidonia oceanica* meadows and all stations were sampled at four depths (Table 1). Posidonia and Enderrocat are shallower (approximately 10 m) than Bahia and Station 4 (30 m). The temporal variability was studied in the oligotrophic coastal Bay of Villefranche in October and November 2002, March and July 2003 at four depths (Table 1). Point B located at the entrance of the bay (43° 41′N; 7°19′E, Fig. 1b) is a long-term monitoring station. All bottle samples were incubated *in situ* during 24 h on a vertical line attached to a floating buoy.

### Hydrography, irradiance and nutrients

Vertical profiles of temperature, salinity and oxygen saturation were obtained at each station using a SeaBird SBE19 CTD in the Bay of Palma or SBE25 in the Bay of Villefranche. Profiles of light penetration were performed with a LI-COR spherical sensor (LI-193SA) connected to a LI-1400 data–logger. Seawater was collected at four depths within the euphotic layer at each station (Table 1) using single 12 l Niskin bottles. Dissolved inorganic nutrients concentrations were measured using automated colorimetric techniques after filtration through Whatman GF/F glass fibre filters^74^. Dissolved silicate was measured after filtration through 0.45 µm polyvinylidene fluoride membrane filters (PVDF, Millipore). Chlorophyll-*a* was measured on GF/F filters that were stored frozen until extraction and analysis by high-performance-liquid chromatography^75^.

### Dissolved organic carbon (DOC)

Due to the low particulate carbon concentrations in the two sites, total organic carbon samples were collected and not filtered in order to minimize the risk of contamination. Samples of DOC concentration were collected in combusted (450 °C for 24 h) glass ampoules (Wheaton), acidified with 85% H_3_PO_4_ flame-sealed immediately after collection and stored in the dark at ambient temperature pending analysis. DOC concentration was measured by high temperature combustion with a Shimadzu TOC-5000 total organic carbon analyser. All concentrations are reported as the average of three replicate injections.

### Picoplankton abundance

The abundances of heterotrophic prokaryotes, *Prochlorococcus* spp, *Synechococcus* spp and photosynthetic picoeukaryotes were estimated using a bench-top flow cytometer (Becton and Dickinson FACScalibur) equipped with a laser emitting at 488 nm. Aliquots of 2 ml were fixed with 1% paraformaldehyde and 0.05% of glutaraldehyde (final concentration), frozen in liquid N_2_ and stored at −80 °C pending analysis. The samples for heterotrophic prokaryotes abundance were previously stained with Syto-13 Molecular Probes (2.5 µM) and an aliquot of 0.96 µm fluorescent latex beads (Polysciences) was also added as internal standard to each tube. The beads were calibrated every day using a beads solution (TrueCount beads) for which the bead abundance is precisely known. Heterotrophic prokaryotes abundance was identified in plots of side scatter (SSC) versus green fluorescence (FL1) as described by^76^. *Prochlorococcus* spp and *Synechococcus* spp were identified in plots of SSC versus orange fluorescence (FL2) and red fluorescence (FL3). PA had a higher SSC and FL3 than both *Prochlorococcus* spp and *Synechococcus* spp and no FL2 signal. In order to convert cell abundance to carbon biomass it was used, the conversion factors proposed by^77^: 12 fg C cell^−1^ for heterotrophic prokaryotes, 1500 fg C cell^−1^ for picoeukaryotes, 32 fg C cell^−1^ for *Prochlorococcus* spp, and 100 fg C cell^−1^ for *Synechococcus* spp. The total phytoplankton carbon biomass was estimated using a carbon-to-chlorophyll-*a* ratio of 120^78^.

### Primary production ^14^C technique

The incorporation of carbon into the dissolved and particulate fractions was measured using the ^14^C technique^79^. Water samples (30 ml) were transferred to one dark and three light bottles and incubated *in situ* at each depth and station. The samples were incubated *in situ* on a vertical line from sunrise to sunset after addition of 30 µCi (1110 Kbq) of sodium bicarbonate (NaH^14^CO_3_). Upon completion of the incubation, two 5 ml aliquots of each sample were placed into 20 ml scintillation vials for determination of total labelled organic carbon (Total Primary Production = DPP + PPP). The unit for DPP and PPP were estimated by the incubation time, which was the daylight time (mg m^−3^ h^−1^). Two more 5 ml aliquots of each sample were filtered through 0.22-µm mixed cellulose esters membrane filters (Millipore) for determination of PPP and the filtrate was collected for determination of DPP. Filters were placed into vials and exposed to concentrated hydrochloric acid fumes for a minimum of 12 h before the addition of 3.5 ml Packard Ultima Gold (PerkinElmer) liquid scintillation cocktail. Liquid samples (total primary production and DPP) were acidified with 100 µl of hydrochloric acid (5N) and bubbled with air for 2 h before addition of 15 ml Ultima Gold XR (PerkinElmer) liquid scintillation cocktail. Radioactivity (dpm) was measured in a Packard Tri-Carb 4000 scintillation counter. Counts from the dark bottles were subtracted from counts measured in the light bottles to correct for non-photosynthetic ^14^C-incorporation. The radioactivity of the NaH^14^CO_3_ added to each sample was estimated from 20 µl aliquots.

### Bacterial metabolism

To estimate *in situ* community respiration, the first set of seawater samples were incubated in dark BOD (Biological Oxygen Demand) bottles (60 ml) on a vertical line *in situ* during 24 h. At each station and depth, 5 bottles were immediately fixed with Winkler reagents^80^ (time 0) and five more bottles were fixed after 24 h. The second set of samples was incubated under dark condition in a temperature-controlled incubator simulating *in situ* temperature to calculate incubator community respiration. The third set of samples was filtered (142 mm diameter, 0.8 µm) to include just the bacterial fraction to estimate the incubator bacterial respiration. The filtration was carried out with a low vacuum pressure system at each station and depth. No more than 10 l of a given sample was filtered through the same filter. The percentage of oxygen consumption in filtered seawater samples compared to incubator whole seawater samples, enabled us to estimate incubator bacterial respiration. This percentage was applied to the *in situ* community respiration to calculate the *in situ* bacterial respiration.

To estimate the effect of filtration on bacterial metabolism, bacterial abundance and production, and DOC concentration were compared at each station and bay between filtered and unfiltered samples at each depth.

To estimate the effect of incubation time on bacterial metabolism, at each station of the Bay of Palma (S4, Posidonia, Enderrocat and Bahia) and depth (surface and bottom) an extra set of filtered and unfiltered seawater samples were incubated in dark BOD bottles (60 ml) during 12, 24 and 48 hours to estimate community and bacterial respiration. For bacterial production rates, seawater samples were incubated in borosilicate bottles on a vertical line *in situ*. At each depth and station, three replicates were recovered at sunrise (0 h of incubation), midday (6 h of incubation) and sunset (about 12 h of incubation depending on the season).

Dissolved oxygen concentration was determined by titration with a potentiometric end-point detection^80^. Analyses to estimate respiration were performed with a redox electrode (9778-SC, Orion) and a custom built titrator. Bacterial production was estimated as the rate of ^3^H-leucine incorporation using standard methods^81^ in Eppendorf vials. Sea water samples were transferred to Eppendorf vials (1.5 ml). Three were spiked with 5 µl of cold leucine (20 nM final concentration) and 25 µl of ^3^H-leucine (10 nM final concentration; Amersham, specific activity 170 µCi nmol^−1^). The fourth sample, to which 100 µl of 100% trichloroacetic acid was immediately added, served as a control.

The conversion factor required to express the incorporation of leucine into carbon units was estimated at stations Station 4 and Enderrocat at the surface (0.5 m) and bottom depth (33 and 16 m, respectively) following the procedure proposed in^82^. A sample (1.8 L) was filtered through a 0.2 µm polycarbonate membrane previously rinsed with milliQ water and with sample water and placed in a bottle with 0.2 l of unfiltered water. The incubation lasted for 2 d in a temperature-controlled incubator simulating *in situ* temperature. Aliquots for bacterial production (leucine incorporation) and abundance (cytometry) were processed every 6 h. The conversion factor (CF) was calculated using the relationship derived by Kirchman and Ducklow in^82^:

$$CF=\mu \frac{{e}^{B}}{{e}^{b}}$$

Where µ is the growth rate (d^−1^) determined from the change in biomass over time, e^B^ is the y-intercept of Ln (biomass in cell ml^−1^) *vs* time and e^b^ is the y-intercept of Ln (leucine incorporation in pmol Leu^−1^l^−1^h^−1^) *vs* time. Four values of conversion factors were obtained and the average (1.15 ± 0.33 kg C mol^−1^; see Table 1 Supplementary Information) was used to convert leucine incorporation to carbon uptake for bacterial carbon demand and growth efficiency (BGE) calculations. BGE was estimated as the ratio of bacterial production to total bacterial carbon demand (BP + BR). A respiratory quotient (RQ) of 1 was assumed^24^.

### Net community production (NCP)

For estimating production of dissolved oxygen, seawater samples (10 replicates) were transferred into transparent BOD bottles (60 ml) and incubated on a vertical line *in situ* during 24 h. At each station and depth, incubations started at sunrise. Five bottles were immediately fixed with Winkler reagents^80^ (time 0) and 5 other bottles were fixed at the following sunrise (24 h incubation). The dissolved oxygen concentration was determined as explained above. NCP was estimated by regressing O_2_ against time.

### Statistical analysis

One-way analysis of variance ANOVA (Statistica 5.0) was performed to evaluate differences among trophic status systems. Variance homogeneity was tested with Cochran’s analysis. The Student-Newman-Keuls test (SNK) was used to discriminate among different treatments after a significant F-test. A Mann-Whitney U test was used when the data were not strictly parametric. The regression model type II was used to evaluate the relationship between variables when both variables were subject to measurement error.

## Supplementary information

Table SI
