# Supplementary material for: Drivers of Microbial Carbon Fluxes Variability in Two Oligotrophic Mediterranean Coastal Systems
Source: Sci Rep. 2019 Nov 27;9:17669. doi: 10.1038/s41598-019-53650-z (PMC6881365; doi:10.1038/s41598-019-53650-z)
Supplement: Supplementary file 1 — Table SI [file 41598_2019_53650_MOESM1_ESM.docx]

Supplementary Information for:

**DRIVERS of microbial CARBON fluxes variAbility in two oligotrophic mediterranean coastal SYSTEMS**

Natalia González-Benítez^1,2,3*^_,_ Lara S. García-Corral^3^, Xosé Anxelu G. Morán^4^, Jack J Middelburg^5,6^, Marie Dominique Pizay^1,2^, Jean-Pierre Gattuso^1,2^.

* Corresponding author: natalia.gonzalez@urjc.es

**Table 1:** Parameters of the four CFs calculated using the relationship derived by Kirchman and Ducklow (1993):

$$CF=\mu\frac{e^{B}}{e^{b}}$$

Where µ is the growth rate (d^-1^) determined from the change in biomass over time, e^B^ is the y-intercept of Ln (biomass in cell ml^-1^) *vs* time and e^b^ is the y-intercept of Ln (leucine incorporation in pmol Leu^-1^l^-1^h^-1^) *vs* time. The average (1.15 ± 0.33 kg C mol^-1^) was used to convert leucine incorporation to carbon uptake for bacterial carbon demand (BCD) and growth efficiency (BGE) calculations.

| **Station** | **Depth** | **µ** | **e^b^** | **e^B^** | **CF** |
| --- | --- | --- | --- | --- | --- |
|  | m | d^-1^ | pmol Leu^-1^l^-1^h^-1^ | cell ml^-1^ | kg C mol^-1^ |
| End | 0.5 | 0.046 | 0.086 | 100583971 | 0.8115 |
| End | 15 | 0.035 | 0.035 | 77641813 | 1.1737 |
| S4 | 0.5 | 0.079 | 0.198 | 172912829 | 1.0398 |
| S4 | 30 | 0.082 | 0.124 | 160671687 | 1.5980 |
